# Costs of postabortion care in public sector health facilities in Malawi: a cross-sectional survey

**DOI:** 10.1186/s12913-015-1216-2

**Published:** 2015-12-17

**Authors:** Janie Benson, Hailemichael Gebreselassie, Maribel Amor Mañibo, Keris Raisanen, Heidi Bart Johnston, Chisale Mhango, Brooke A. Levandowski

**Affiliations:** Ipas, Chapel Hill, NC USA; Ipas, Nairobi, Kenya; Ipas, Durham, NC USA; Ipas, Baltimore, MD USA; Reproductive Health and Rights consultant, Geneva, Switzerland; College of Medicine, University of Malawi, Blantyre, Malawi; Department of Family Medicine, SUNY Upstate Medical University, 475 Irving Avenue, Suite 200, Syracuse, NY 13210 USA

**Keywords:** Abortion, Postabortion care, Cost, Dilation and curettage, Health system, Manual vacuum aspiration, Misoprostol, Malawi

## Abstract

**Background:**

Health systems could obtain substantial cost savings by providing safe abortion care rather than providing expensive treatment for complications of unsafely performed abortions. This study estimates current health system costs of treating unsafe abortion complications and compares these findings with newly-projected costs for providing safe abortion in Malawi.

**Methods:**

We conducted in-depth surveys of medications, supplies, and time spent by clinical personnel dedicated to postabortion care (PAC) for three treatment categories (simple, severe non-surgical, and severe surgical complications) and three uterine evacuation (UE) procedure types (manual vacuum aspiration (MVA), dilation and curettage (D&C) and misoprostol-alone) at 15 purposively-selected public health facilities. Per-case treatment costs were calculated and applied to national, annual PAC caseload data.

**Results:**

The median cost per D&C case ($63) was 29 % higher than MVA treatment ($49). Costs to treat severe non-surgical complications ($63) were almost five times higher than those of a simple PAC case ($13). Severe surgical complications were especially costly to treat at $128. PAC treatment in public facilities cost an estimated $314,000 annually. Transition to safe, legal abortion would yield an estimated cost reduction of 20 %-30 %.

**Conclusions:**

The method of UE and severity of complications have a large impact on overall costs. With a liberalized abortion law and implementation of induced abortion services with WHO-recommended UE methods, current PAC costs to the health system could markedly decrease.

## Background

In Sub-Saharan Africa, health system costs to treat unsafe abortion complications range from $68 to $76 million per year [1]. Health systems could obtain significant cost savings by providing safe abortion care rather than providing often expensive treatment for complications of unsafely performed abortions. Complications from unsafe abortion are unnecessary, preventable and have straightforward technical and clinical solutions. A cost modeling study found that the mean health facility cost per case of unsafe abortion complications in Uganda could be reduced by 43 % with: 1) a shift from dilation and curettage (D&C) to WHO-recommended vacuum aspiration for uterine evacuation (UE) with light sedation and 2) increased percentage of postabortion care (PAC) provided by mid-level providers at lower-level health centers. A shift to safe, elective abortion with recommended technology by mid-level providers was estimated to be seven times less expensive than hospital-based PAC with physicians in a restrictive legal environment in the study [2].

PAC treatment cost estimates in the health care system are affected by the severity of cases and the number of women needing treatment, which is related to the access of legal, safe abortion in a country [3]. In Malawi, where abortion is currently legal only to save the life of a pregnant woman, an estimated 67,300 induced abortions occur annually, with approximately 29,500 women treated in all health facilities and 18,600 treated in public facilities for PAC [4, 5]. One in five women presenting at health facilities for PAC are treated for severe abortion complications, such as hemorrhage and sepsis, indicating a high level of required management and associated health system costs [4, 6].

Cost savings are useful to policymakers who face increasing health care needs while managing increasingly limited budgets, but current data are needed [6]. The most recent study of PAC costs in Malawi collected data from a small sample of health facilities in 1998. The per-case cost of treating postabortion complications ranged from $30 in mission hospitals to $42 in public hospitals. Clients paid an estimated extra $8 in user fees, travel and other costs [7]. A literature review on PAC costs in Africa showed that as countries switch from mainly D&C to manual vacuum aspiration (MVA), overall costs decrease due to decreases in length of hospital stay, waiting time, staff time spent with patient and treatment time [8]. As over half of all women treated for PAC in Malawi in 2009 received treatment by MVA, overall health system costs of PAC treatment may have decreased since the original study 15 years ago, or increased due to greater access to health facilities providing PAC and more women seeking care [6].

This study: 1) describes currently used PAC treatment categories; 2) estimates per-case and annual health system costs of treating complications from unsafe abortion; and 3) documents potential health system savings by shifting from providing treatment for unsafe abortion complications to providing safe, legal induced abortion using WHO-recommended methods in Malawi.

## Methods

Cost estimates were calculated from three data sources: 1) an original survey completed by one or more PAC providers working at a public health facility to determine the personnel, supplies and medications involved in typical PAC treatment; 2) national and international reports of salary and supply/drug costs; and 3) a prior national abortion morbidity study that provided the number of PAC cases and their distribution of complications severity in PAC-providing health facilities in Malawi. Facility survey data and cost information were analyzed in *Savings*, an Excel-based tool to calculate per-case costs [2]. Cost calculations were generated for different caseload scenarios and were scaled up to create national estimates of health system costs for abortion complications.

The study was approved by the Institutional Review Board of the University of Malawi College of Medicine, government representatives and the directors of each facility. All provider-data collectors signed an informed consent form. No names, record numbers or other identifying information were collected on individual patients.

PAC treatment data were collected in 15 purposively-selected facilities, selected in consultation with the Reproductive Health Unit (RHU) of the Malawi Ministry of Health. Facility selection criteria included public sector facilities that treat abortion complications, and location in urban and rural areas of the three main geographic regions, Central, Northern and Southern. The sample included four tertiary hospitals, five secondary hospitals, and six primary health centers, representing 16 % of PAC-providing public facilities in Malawi, and 40 % of the estimated PAC caseload in public facilities in 2009 [6].

A data collection tool from a similar study in Nigeria was adapted and pre-tested for use in Malawi [9]. Following training, providers at each of the study facilities, in consultation with fellow providers at that site, completed the survey to record typical treatment practices for PAC cases in their locations. Data were collected between May-June 2010.

The survey was implemented to capture recurrent costs of abortion care and included detailed information on the kinds, quantities and costs of medications and supplies needed for typical PAC treatment categories (UE of incomplete abortion with no additional surgical interventions and UE with surgical procedures) and UE procedure types (D&C, vacuum aspiration (primarily MVA) and misoprostol-alone) available at the facilities. The survey also captured the amount of time spent by various cadres of health care personnel at each step of clinical care for the PAC treatment and UE types. Data on indirect costs was not included due to concerns about data quality and the study’s focus on recurrent costs most amenable to policy change.

Salaries by cadre and costs of medications and supplies were obtained primarily from the RHU and the 2009–10 Central Medical Stores (CMS) Catalogue. If a supply cost was not available from the RHU nor CMS, an average of cost reported in the facility surveys was used. WHO’s 1994 Mother-Baby Package spreadsheet and 1999 documentation also supplemented information on cost for a unit of blood and amounts of time expended to treat septic women [10, 11]. All national costs were based on 150 Malawi Kwacha (KWK) to 1 USD for the 2010 data collection period (Table 1).

**Table 1:** Monthly caseload distribution by treatment category and procedure type in 93 PAC-providing public health facilities (*n* = 1,207 cases)^1^

	MVA		D&C		Misoprostol		Overall	
	*n*	%	*n*	%	*n*	%	*n*	%
**PAC treatment category**								
**Simple**	**582**	**87 %**	**427**	**80 %**	**3**	**100 %**	**1,012**	**84 %**
UE with associated treatment	582		427		3		1,012	
**Severe non-surgical**	**84**	**13 %**	**108**	**20 %**	**0**		**192**	**16 %**
UE with treatment of sepsis	61		74		-		135	
UE with blood transfusion	16		24		-		40	
UE with treament of sepsis & blood transfusion	7		10		-		17	
**Severe surgical**	**1**	**<1 %**	**2**	**<1 %**	**0**		**3**	**<1 %**
UE with surgical repair & treatment of sepsis	1		1		-		2	
UE with surgical repair & blook transfusion	-		-		-		-	
UE with surgical repair, treatment of sepsis & blood transfusion	-		1		-		1	
**All procedures**	**667**		**537**		**3**		**1,207**	

^1^Caseload data collection occurred in 2009

PAC caseloads, diagnosis, treatment and complications severity data for all 93 PAC-providing public facilities were extracted from a separate, nationally-representative prospective study on abortion morbidity conducted in Malawi in 2009 (detailed methodology provided elsewhere) [4, 6]. Of the 542 PAC cases treated in the 15 facilities during the morbidity study’s data collection period, 41 (8 %) were excluded from subsequent analysis due to missing UE procedure information.

Analysis was structured by three categories of PAC treatment for each UE type: 1) PAC with UE and associated treatment such as administration of pain medications, antibiotics and/or fluids (simple); 2) PAC with UE and blood transfusion and/or sepsis treatment (severe non-surgical); and 3) PAC with UE and surgical repair such as interventions to repair the cervix, uterus or internal organs (severe surgical).

Facility survey data and cost information were entered into *Savings*. Unit costs of each input (supplies, medications, personnel) were multiplied by their respective amounts used for each PAC treatment regimen and UE procedure type. The cost of each input’s contribution were then summed to estimate the overall per-case cost of each treatment regimen by UE procedure for each facility.

Data from the *Savings* calculations were transferred to Stata versions 11–12 (StataCorp, College Station, TX US) to generate median per-case costs of each PAC treatment regimen by UE procedure type at each level of care. To estimate the annual cost of PAC in public facilities, median per-case costs were applied to their respective proportion of annual caseload from the 93 PAC-providing public facilities in the national abortion morbidity study. We assumed all public facilities had the same PAC treatment practices as the 15 surveyed facilities. Interquartile and overall ranges were calculated for each combination of inpatient/outpatient, UE type, and complication type. The 25 % and 75 % percentile of each treatment category’s per-case cost were applied to their respective caseloads and summed to obtain an interquartile range estimate for the total annual cost (USD).

Using national cost estimates of PAC and safe abortion care, we modeled two hypothetical scenarios for the long-term effect of adoption of broader indications for legal abortion where: 1) only MVA is available; and 2) 70 % of procedures are performed with MVA and 30 % with misoprostol. These scenarios were chosen due to the current availability of these two WHO-recommended UE methods in Malawi. Several international experts in abortion care provided estimates of the kinds and amounts of supplies and medications and the time necessary for a trained midwife to perform a safe, first-trimester induced abortion on an outpatient basis for the two methods. Costs of each input were applied to create a per-case cost for each scenario. We assumed the annual number of women seeking PAC in public facilities remained the same; women were provided with first-trimester induced abortion instead of PAC; and procedures were performed using mild sedation (for MVA) or analgesics (for misoprostol-alone) for pain management.

## Results

A large majority of PAC cases (84 %) in the 93 PAC-providing public facilities were simple cases, requiring UE and associated treatment (Table 1). Sixteen percent of women were treated for severe complications that did not require surgery. MVA was used for UE in 55 % of all cases, while 44 % were treated with D&C. A larger proportion of cases with severe non-surgical complications were treated with D&C (56 %) versus MVA (44 %). Conversely, simple cases were more frequently treated with MVA (58 %) than D&C (42 %). Misoprostol use was negligible.

Overall, the median per-case costs for labor and supply inputs for simple UE were approximately $7 each (Table 2). Within the study facilities, the estimated median per-case cost of treatment for all PAC cases was $40 (Table 3). The median cost per D&C case ($63) was 29 % higher than an MVA case ($49) for every PAC treatment category (Table 3). This difference was especially marked for simple cases as the median cost of care for a simple PAC case with D&C, $19, was 46 % higher than with MVA ($13) and 58 % higher than misoprostol ($12).

**Table 2:** Estimated median per-case costs (USD) for labor and supply components for simple uterine evacuation by treatment type in 15 PAC-providing public health facilities^1^

	MVA	D&C	Misoprostol	Overall
PAC input costs				
Labor inputs	$7	$7	$7	$7
Supply and medication inputs	$6	$14	$4	$7

^1^Cost data collection occurred in 2010. Cost data on uterine evacuation type was only available from facilities providing each type of treatment. Data on MVA, D&C, and misoprostol was available from 15, 9 and 2 facilities, respectively

**Table 3:** Estimated median per-case costs (USD) by treatment category and procedure type in 15 PAC-providing public health facilities (*n* = 501 cases)^1^

	MVA	D&C	Misoprostol	Overall
	*n* = 280	*n* = 220	*n* = 1	*n* = 501
	15 facilities	9 facilities	2 facilities	15 facilities
**PAC treatment category**				
**Simple**	**$13**	**$19**	**$12**	**$13**
UE with associated treatment	$13	$19	$12	$13
**Severe non-surgical**	**$61**	**$67**	-	**$63**
UE with treatment of sepsis	$46	$52	-	$46
UE with blood transfusion	$52	$58	-	$52
UE with treatment of sepsis & blood transfusion	$85	$91	-	$85
**Severe surgical**	**$119**	**$138**	-	**$128**
UE with surgical repair & treatment of sepsis	$119	$119	-	$119
UE with surgical repair & blood transfusion	-	-	-	-
UE with surgical repair, treatment of sepsis & blood transfusion	-	$158	-	$158
**All procedures**	**$49**	**$63**	**$12**	**$40**

^1^Cost data collection occurred in 2010

The estimated median per-case cost also varied by treatment category. Treatment of a case with severe non-surgical complications ($63) was almost five times higher than that of a simple PAC case ($13). Although few in number, cases with severe surgical complications were especially costly to treat ($128), almost 10 times higher than a simple case. Even within the same UE procedure type, the more severe the complications, the higher the estimated per-case cost. For women treated with MVA, the cost of a severe non-surgical case was almost five times higher than that of a simple case.

Almost 15,000 women were treated for abortion complications requiring UE procedures in all 93 PAC-providing public health facilities in Malawi in 2009, at an estimated cost of $314,008 [Interquartile range $287,406-$502,522] (Table 4). While cases of severe non-surgical and surgical treatment represent only 16 % of all PAC cases treated in the facilities, their related costs consume nearly one-half (49 %) of overall costs.

**Table 4:** Estimated current annual cost (USD) of PAC in 93 PAC-providing public health facilities^1^

	Median per-case cost	Caseload^2^	Annual cost (USD)	Annual cost (MWK)^3^
**PAC treatment category**				
Simple *[interquartile range]*	$13 *[$12 - $25]*	12,346	$161,738 *[$141,984 - $307,425]*	24,260,676
Severe non-surgical *[interquartile range]*	$63 *[$61 - $81]*	2,342	$147,571 *[$141,949 - $189,969]*	22,135,680
Severe surgical *[interquartile range]*	$128 *[$95 - $140]*	37	$4,699 *[$3,473 - $5,128]*	704,916
**All procedures** *[interquartile range]*	**$40** *[$36 - $55]*	**14,725**	**$314,008** *[$287,406 - $502,522]*	**47,101,272**

^1^Caseload data collection occurred in 2009; cost data collection occurred in 2010

^2^Annual caseload calculated as 1 month of PAC cases requiring UE times 12.2 (unweighted data)

^3^Malawian Kwacha: 150 MWK = 1 USD

In the 93 PAC-providing public facilities, two potential scenarios in which treatment of abortion complications is shifted to legal, safe abortion both result in substantial annual cost savings for Malawi (Table 5). The first scenario, in which all women seeking first-trimester induced abortion choose MVA, result in an estimated cost reduction of 20 %. The second scenario, with both MVA and misoprostol available, yields an estimated 30 % reduction.

**Table 5:** Estimated costs (USD) of shifting from PAC to safe, legal induced abortion in 93 PAC-providing public health facilities^1,2^

	Current	Projected	
		Only MVA^3^	MVA/Misoprostol^4^
**Procedure type distribution for first trimester, legal, induced abortion**			
MVA ($17)		100 %	70 %
Misoprostol-alone ($10)		-	30 %
**Total annual cost**	**$314,008** *[$287,406 - $502,522]*	$250,332	$219,408
**Percent decrease from current costs**	-	**20 %**	**30 %**

^1^Cost data collection occurred in 2010

^2^All costs were calculated using median per-case costs

^3^Women only have MVA available

^4^Women have a choice between MVA and misoprostol-alone

## Discussion

The majority of PAC cases were treated for simple, incomplete abortion while surgical treatment was rare. A sizeable proportion of cases were treated with D&C, an outdated technique being replaced by MVA and medical abortion worldwide [12]. Providers appeared to select D&C for more intensive treatment, despite the fact that MVA and misoprostol are appropriate for UE in PAC cases of any treatment category. MVA and misoprostol use have been shown to be safe and effective in outpatient settings when performed by trained physician or mid-level providers, and resulting in shorter length of patient stay and lower care costs [2, 9, 13–16].

The method of UE and severity of complications had a large impact on overall costs. The per-case cost was markedly higher for D&C than MVA, even for cases within the same treatment category of complications, due to increased supplies and medication inputs for D&C procedures. While very few women were being treated for PAC with misoprostol-alone, the median per-case cost of this treatment was the lowest of all three methods, indicating that increased use of misoprostol would reduce overall treatment costs. Our data also confirmed other research findings that increased severity of complications are associated with increased costs of treatment [9, 17, 18].

Treating 18,600 women for PAC in public health facilities annually represents a significant and preventable burden on the public health system [4]. The median cost of treating one PAC case in public facilities, $40, is markedly higher than the 2011 per capita spending by the Malawi government on health, $23 [19]. A liberalized abortion law and access to safe abortion in public health facilities yielded a 20-30 % decrease in current PAC costs. This drop would occur even though a high-quality first-trimester legal abortion with MVA (with appropriate use of supplies and pain management drugs) costs somewhat more than the current treatment of a simple PAC case.

Our study had the advantage of drawing from two data sources for caseload and costs collected within about a year of each other. The 2009 abortion morbidity study was a large, nationally-representative study of abortion treatment in health facilities, in which standardized information on abortion caseload was prospectively captured. These comprehensive caseload data increased the validity of our cost estimates.

To our knowledge, only two other studies have estimated national costs of PAC using nationally-representative, prospectively-collected information on the number and clinical care details of women presenting for treatment of abortion complications in health facilities [17, 20]. Other cost estimates have relied on PAC caseloads from key informant estimates or facility records as part of national studies on abortion incidence or smaller facility studies, methodologies subject to recall bias and underreporting [9, 18, 21–23].

Limited resources and the need for in-depth data collection on complete resources used to treat various levels of PAC complications led us to select fewer facilities for the in-depth survey. We aimed for representativeness by purposively selecting facilities at primary through tertiary levels and in rural and urban locations, including four tertiary hospitals which treat almost one-half of PAC cases seen in public facilities in Malawi. Provider estimation of medications, supplies and personnel time needed to treat PAC in public health facilities is likely reasonable due to their experience providing this care.

Annual costs are likely underestimates due to exclusion of certain cases or types of costs. Women with complications of unsafe abortion were excluded from the study if they were: 1) treated without involving UE; 2) referred elsewhere; or 3) unable to access care in the public sector due to transport, perceived or real cost and/or other barriers. Private and non-governmental facilities play an important role in PAC service delivery in Malawi but were excluded from our estimates.

We have presented recurrent costs of public service delivery, specifically, for personnel time and use of medications and supplies, inputs which are most amenable to improvements in safe abortion provision. Start-up costs such as provider training and supervision, and overhead or indirect costs were not included in our calculations. Including patient accommodations and fees, facility overhead, and amortized capital costs significantly add to overall costs but will not be influenced by type of abortion care treatment used.

Changes in service delivery from PAC to safe abortion may not result in reduced overall expenditures within health systems, but instead could improve the efficiency of personnel time and reduce the need for complex clinical treatments, increasing available resources for other critical reproductive and maternal health needs. Furthermore, the benefits of legal abortion extend well beyond reduced health system costs, even with potential shifts in abortion caseloads from clandestine, unsafe environments to safe services in public hospitals and health centers. Improvements in women’s health and human rights, saving their lives, and strengthening their families must be prioritized [24, 25].

## Conclusions

Our findings show the cost benefits to health systems that could result from legal and service delivery reform. These advantages are particularly critical in light of limited resources to support growing demands for health care in Malawi and throughout Sub-Saharan Africa. Equally important, policy and practice changes that expand women’s access to safe abortion yield improvements in women’s lives, health and reproductive rights.

## Abbreviations

CMS: Central medical stores catalogue
D&C: Dilation and curettage
IQR: Interquartile range
KWK: Malawi Kwacha
MVA: Manual vacuum aspiration
PAC: Postabortion care
RHU: Reproductive Health Unit
UE: Uterine evacuation
USD: United States dollar
WHO: World Health Organization
